# Dr. Langslow, (in Explanation) upon Apoplexy

**Published:** 1802-01

**Authors:** R. Langslow

**Affiliations:** Halesworth, Suffolk


### Dr. Langslow, *(in Explanation) upon Apoplexy.*

### To the Editors of the Medical and Physical Journal.

Gentlemen,

SINCE the publication of your laſt Number, a Report has been circulated in this neighbourhood, either from a miſconception of my reaſoning, or from a worſe motive, That in my Anſwer to Mr. Crowfoot upon Apoplexy, I have intended to aſſert, that *no* caſe of that diſeaſe ever *did* or *can* occur without extravaſation of *blood* upon the brain.* Now, I truſt that no candid inveſtigator of the doctrines there advanced will diſcover any *real* ground for ſuch a report.

That I conceive "*preſsure upon the brain*" to be the general cauſe of Apoplexy is true; and it is equally ſo, that I am of opinion the compreſſing fluid is ſometimes (particularly in thoſe caſes which terminate without leaving paralyſis) *ſerous.* That a very large proportion of the fatal caſes of Apoplexy are occaſioned by the effuſion of red blood from *vaſcular rupture* in the brain, I think there is little doubt; indeed, repeated diſſections confirm this idea; I have myſelf, in the courſe of thirty years pretty extenſive practice, inſpected many, and generally found blood, in ſuch unfortunate inſtances, the compreſſing cauſe: And this opinion is ſtill farther corroborated by the experience of the truly ingenious Dr. G. Fordyce, who aſſures me, he has found this to be the ſource of Apoplexy ſo generally, as to induce him to think it rarely ariſes from any other cauſe.† But, on

---

* Upon referring to Mr. C's ſtatement, I obſerve that he ſays, upon my return from the ſick chamber, &c. &c. to the parlour, "that I declared there was *conſiderable* extravaſation of *blood* upon the brain, &c. &c." Now the parties who were preſent at that converſation, aſſert that they did not *hear me* uſe either the word *conſiderable* or *blood* upon that occaſion. From this circumſtance, and the expreſſion being directly repugnant in its import to my *opinion*, I think myſelf juſtified in denying this part of Mr. C's paper; nay, I never did conſider the extravaſation as *conſiderable* in this inſtance, nor did *I ever* think the compreſſing fluid *blood*, which was my motive for laying ſo much *ſtreſs* on the term *exudation*, at our ſubſequent altercation upon the ſubject, when Mr. C. thought proper to declare the *two* words conveyed a diſtinction without a difference; but it will ſurely be allowed, that *increaſed exudation* from the exhalants, and effuſion from a ruptured blood-veſſel, may prove extremely different in their ſubſequent effects upon the ſyſtem, notwithſtanding the general term *extravaſation* would apply with equal propriety in *both* caſes.

† He informs me the late celebrated anatomiſt, Mr. Cruikſhanks, died of apoplexy; that he was preſent at the diſſection of the brain, and found extravaſation of *blood* the cauſe of his death.

on the other hand, ought it not to be confidered, that as *all* the cafes in which the brain has been examined by Dr. G. F. and myfelf, the Apoplexy ended in death; it will by no means follow, that the difeafe, when terminating favorably, *must necessarily* have been produced by an exciting caufe *exactly* fimilar? Nay, I am inclined to believe, and I fhall probably be fupported in *such* an opinion by many of your Readers, that in fome of thofe cafes where the functions of the brain are *partially* reftored in a few hours, after the compreffing caufe has been applied, and the abforbents have *so speedily* been enabled to *diminish* the compreffing caufe, that in *such instances* the fource of preffure has probably been the exudation of *serous fluid* driven through the orifices of the exhaling arteries. And I conceive it is by no means unlikely, this was the cafe with the patient whofe apoplectic attack has been the fource of the prefent controverfy, as the fymptoms of preffure on the brain began to decline in about twenty minutes, or half an hour, after fhe was bled; which (fuppofing the caufe to be exuded ferum) was time fufficient to admit of the abforbents taking up *such* a portion of the extravafated fluid, as would allow *a partial* reftoration of the fenforial functions. That the adminiftration of a vomit, in *such* a cafe, would have been feverely cenfured by a large majority of your Readers, I have no doubt; and I feel it incumbent upon me before I conclude, to ftate, that if I find what I have already written upon this important fubject honoured with the approbation of yourfelves and numerous Correfpondents, that I fhall be encouraged, at fome future period, when lefs urgently preffed by profeffional engagements, to trouble you with fome farther Obfervations upon the Caufe and Treatment of this very alarming and dangerous malady; when I fhall expect to experience but little difficulty in making it appear, that the fuddenly fatal termination of fome cafes of Apoplexy which have formerly occurred in this county, did probably arife from a treatment *founded* upon the following ftrange hypothefis,— "That the Caufe of Apoplexy exifts in, or originates from, the Stomach, and that Vomits are, *consequently*, the beft, moft efficacious, and judicious Remedies in *such Cases!!!* the affections of the brain being merely fymptomatic, or a fecondary effect." It will be ufelefs to point out to the generality of your Readers, the fatal and dangerous tendency of fuch a doctrine, or the urgent neceffity for its *immediate* refutation; I fhall, therefore, fanguinely hope for your and their affiftance in my endeavours to accomplifh this humane and benevolent purpofe. With apologizing for my long trefpafs on your time and paper both both in the laſt and preſent Number of your truly valuable and uſeful Journal, I ſubſcribe myſelf,

Yours, &c.

R. LANGSLOW, M.D.

*Haleſworth, Suffolk, Dec.* 10, 1801.

P. S. The provincial paper of this week announces the publication of a pamphlet, entitled, "Obſervations on the Opinion of Dr. Langſlow, that Extravaſation is the general Cauſe of Apoplexy." Report ſtates this work to be the joint production of Dr. Girdleſtone and Mr. Crowfoot. I have not yet ſeen it; but a Phyſician being *said* to have had a hand in compoſing it, I ſhall feel myſelf bound to pay it *immediate* attention: And if I find the arguments it contains are intended to ſupport Mr. Crowfoot's opinion, "That the general cauſe of Apoplexy *is not* extravaſation or *pressure on the brain*, I ſhall expect but little difficulty in refuting them.

---

*Extract from the Ipswich Paper.*

*Haleſworth, Dec.* 13, 1801.

### APOPLEXY.

Dr. LANGSLOW thinks it neceſſary to ſtate, he did not ſee Mr. Crowfoot's Obſervations on Apoplexy till this day, or this card would have appeared in laſt week's paper. He there finds the report circulated of Dr. Girdleſton's *supporting* Mr. C's theory of the cauſe of Apoplexy *confirmed*, (i. e. "That preſſure on the brain *is not* the general cauſe of Apoplexy, but that it exiſts in, or originates from, the ſtomach, and therefore vomits are the beſt and moſt judicious remedies in ſuch caſes ! ! !")

Dr. L. conſequently *now* conſiders himſelf at iſſue with thoſe two gentlemen, upon this truly important pathological queſtion; and wiſhes *the future* claims of the reſpective parties to public favour and patronage may *rest* upon the *decision*. Dr. L. had ſent (before he ſaw Mr. C's book) a ſecond paper to the Medical and Phyſical Journal for the next month, and which he hopes will appear.

N. B. An anſwer to the Obſervations will be publiſhed as ſpeedily as the profeſſional avocations of Dr. L. will admit; and he truſts the candid reader, who may have been influenced by Mr. C's reaſoning, will ſuſpend his opinion on the practical merits of this production for a ſhort time.

CRITICAL

